# Seasonal Dynamics of Metabolites in Needles of *Taxus wallichiana* var. *mairei*

**DOI:** 10.3390/molecules21101403

**Published:** 2016-10-20

**Authors:** Li Yang, Zan-Sheng Zheng, Fang Cheng, Xiao Ruan, De-An Jiang, Cun-De Pan, Qiang Wang

**Affiliations:** 1 Ningbo Institute of Technology, Zhejiang University, Ningbo 315100, China; yangli0817@yeah.net (L.Y.); chengfang@163.com (F.C.); ruanxiao@nit.net.cn (X.R.); 2 College of Life Sciences, Zhejiang University, Hangzhou 310058, China; dajiang@zju.edu.cn; 3 Ningbo Chemgoo Pharmaceutical Technology Innovation Limited, Ningbo 315112, China; willzheng@chemgoo.com; 4 College of Forestry and Horticulture, Xinjiang Agricultural University, Urumqi 830052, China; pancunde@163.com

**Keywords:** *Taxus wallichiana* var. *mairei*, metabolites, seasonal dynamics, meteorological parameters

## Abstract

Seasonal variations of the phytochemicals contents in needles of *T. wallichiana* var. *mairei* due to the effects of growth meteorological parameters were investigated in this study. The needles of *T. wallichiana* var. *mairei* were collected from different months and the contents of taxoids (paclitaxel, 10-deacetylbaccatin III (10-DAB), baccatin III, cephalomannine, 10-deacetyltaxol (10-DAT)), flavones (ginkgetin, amentoflavone, quercetin) and polysaccharides were quantified by ultra performance liquid chromatography (UPLC) and the resonance light scattering (RIL) method. The content of taxoids gave the highest level of 1.77 ± 0.38 mg·g^−1^ in January, and the lowest value of 0.61 ± 0.08 mg·g^−1^ in September. Unlike taxoids, the content of flavonoids was the highest in August. The content of polysaccharides reached peak value of 28.52 ± 0.57 mg·g^−1^ in September, which was two times higher than the lowest content of 9.39 ± 0.17 mg·g^−1^ in January. The contents of paclitaxel, 10-DAB, 10-DAT and polysaccharides significantly depended on meteorological parameters. The mean of minimum temperature (R = −0.61) and length of daylight (R = −0.60) were significantly correlated to 10-DAB content, while 10-DAT level showed significant correlation with length of daylight (R = −0.70) and relative humidity (R = 0.70). In addition, temperature had significantly negative effect on the content of paclitaxel and a significantly positive effect on that of polysaccharides. This study enriched the knowledge on the accumulation pattern of metabolites and could help us to determine the collecting time of *T. wallichiana* var. *mairei* for medicinal use.

## 1. Introduction

*Taxus wallichiana* var. *mairei*, also known as the Chinese yew belonging to the Taxaceae family, is a protected, valuable and natural anti-cancer plant endemic to China [1]. The extracts of the plant have been commonly used in traditional Chinese medicine for cancer treatment [2,3,4]. As one of the most broad-spectrum anticancer agents, paclitaxel has been proved to have a remarkable effect against breast, lung, blood, and ovarian cancers [5,6,7]. Since the discovery of paclitaxel with its significant anticancer biological activity in the bark of *T. wallichiana* var. *mairei*, extensive efforts have been taken to identify other members of the taxoids group exhibiting potential anti-tumor activities. In relation to this, 10-DAB and baccatin III as paclitaxel precursors can be converted to paclitaxel or taxotere, a chemically modified analogue with more efficient anti-cancer activity than paclitaxel [8]. Thus far, more than 500 taxoids have been identified, some of them have exhibited significant clinical effects [9,10,11], and new taxoids continue to be isolated from the needles, bark, stems, and roots of *T. wallichiana* var. *mairei*.

Except for taxoid terpenoids, there also are many kinds of bio-active substances in *T. wallichiana* var. *mairei*, such as polysaccharides and flavones [12,13,14]. Although it is well known that polysaccharides play an important role in the growth and development of living organisms, they have not been extensively explored in *Taxus* species as major bio-active components [15]. Recently, the discovery and evaluation of polysaccharides have drawn more attention for their biological activities such as antitumor, anti-cardiovascular disease, antioxidant activities as well as the ability to promote immunologic function [4,15,16,17,18,19]. Flavonoids, a large category of plant polyphenol secondary metabolites [12,20], displayed wide pharmacological activities, including anti-leukemic, anti-oxidative, anti-inflammatory and immunomodulatory effects [21,22,23]. To date, many flavonols and biflavones have been isolated from *Taxus* species, such as sciadopitysin, kaempferol-4′-methyl ether, 7′′-*O*-methylamentoflavone, quercetin, amentoflavone, and ginkgetin [12,24,25].

Genetic, physiological and environmental factors contribute to the overall phytochemical profile of plants [26,27,28,29]. It is generally known that external factors (temperature, humidity, precipitation and ultraviolet radiation) affected some processes associated with the growth and development of the plant, even influencing the ability to synthesize secondary metabolites, resulting in the change of the overall phytochemical profile that plays a strategic role in the production of bioactive substances [29]. Thus, the production of phytochemical components critically depends on environmental conditions, and the yields in plants gathered from different seasons might be discrepant. The variability of taxoids in *T. wallichiana* var. *mairei* has been reported in previous studies. The seasonal variation of phytochemical content in the branches and leaves of *Taxus wallichiana* var. *mairei* showed that the peak level of paclitaxel and cephalomannine content appeared in May, and the highest values of baccatin III and 10-deacetylbaccatin III were in September and April, respectively [30]. The effects of growth climate conditions for paclitaxel and other derivatives indicated that the contents were significantly affected by light intensity, humidity and soil quality [31]. In addition, strong sun and drought were favored for the yield of paclitaxel content [32]. However, except for paclitaxel, knowledge of other taxoid flavones and polysaccharides accumulation in *T. wallichiana* var. *mairei* with seasonal variation is still limited. In this work, we collected the plant materials twice a month and the data of meteorological parameters, analyzed the content of taxoids, flavonoids and polysaccharides, and established the correlation between phytochemical content and meteorological parameters. It could help us to determine the optimal collecting time of *T. wallichiana* var. *mairei* for medicinal use.

## 2. Results and Discussion

### 2.1. Seasonal Variation of the Taxoid Content

The total content of five taxoids ranged from 1.77 ± 0.38 to 0.61 ± 0.08 mg·g^−1^ and was significantly higher in the winter season from December to February (Table 1). The highest content emerged in January, and the lowest value in autumn September, respectively. The previous studies proved that the highest level of paclitaxel and derivatives appeared in winter [33,34]. In addition, a seasonal dynamic pattern of individual taxoid content was also observed. The highest levels of 10-DAB, baccatin, cephalomannine and paclitaxel (0.76 ± 0.06, 0.78 ± 0.22, 0.18 ± 0.03 and 0.40 ± 0.08 mg·g^−1^) were detected in winter, except that the peak level of 10-DAT (0.26 ± 0.03 mg·g^−1^) appeared in summer. The lowest levels of baccatin, cephalomannine, 10-DAT and paclitaxel were observed in September. In the present work, the content of 10-DAB was higher than that of paclitaxel during the same year, which was similar to the recent reports about the analysis of taxoids in *T. brevifolia* [35] and *T. baccata* [36].

**Figure 1:**
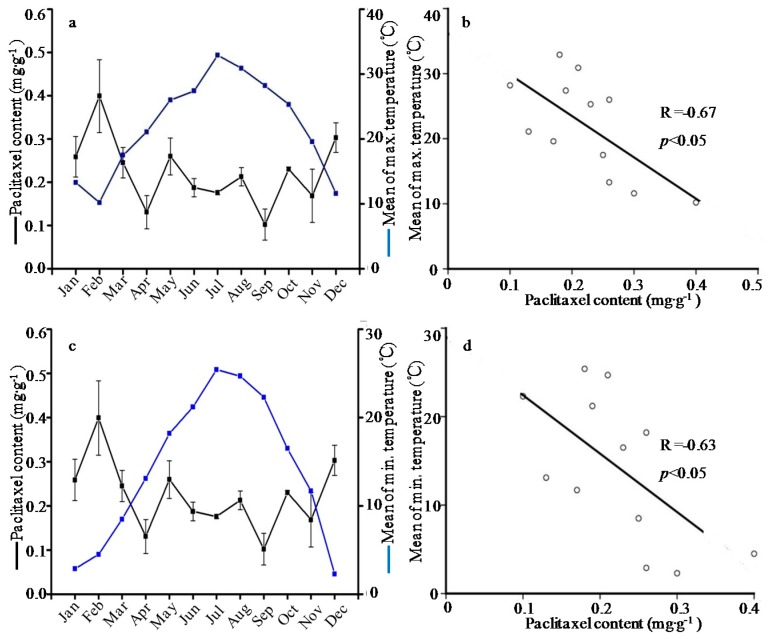
Relationship (**a**) and correlation (**b**) between paclitaxel content and mean of maximum temperature; and relationship (**c**) and correlation (**d**) between paclitaxel content and mean of minimum temperature.

**Figure 2:**
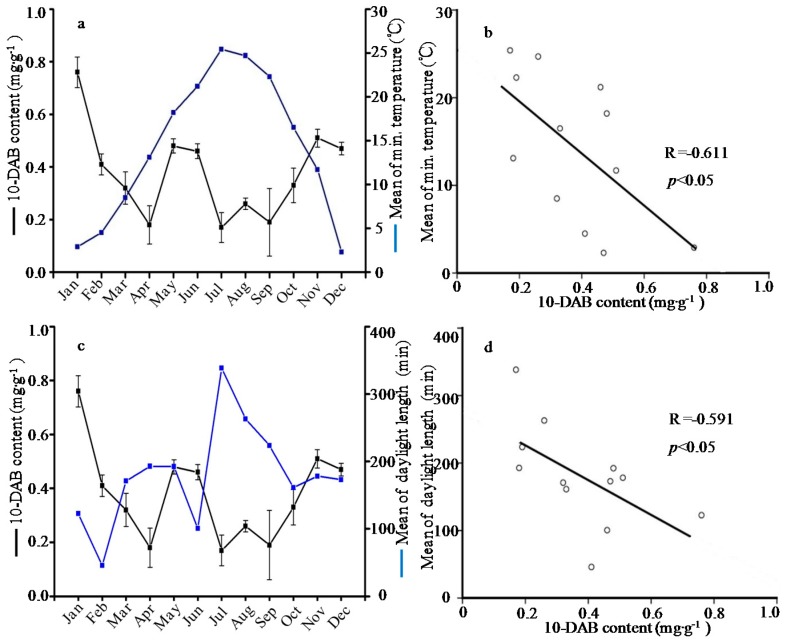
Relationship (**a**) and correlation (**b**) between 10-deacetylbaccatin III (10-DAB) content and mean of minimum temperature; and relationship (**c**) and correlation (**d**) between 10-DAB content and mean of daylight length.

**Figure 3:**
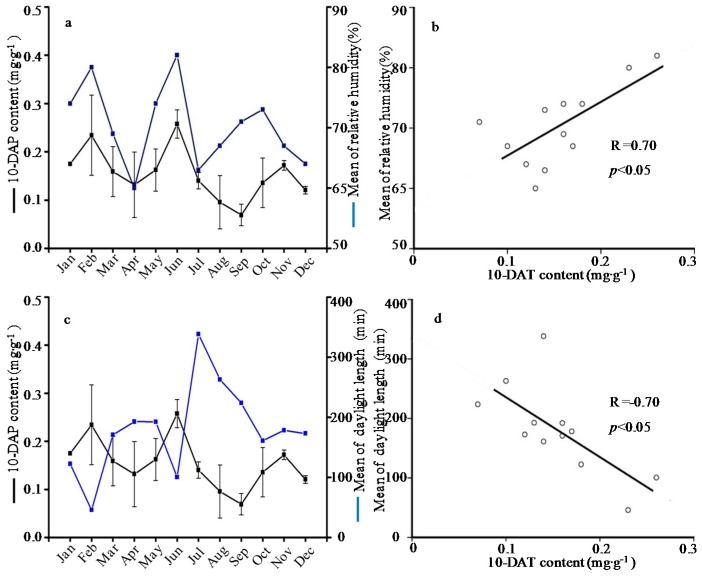
Relationship (**a**) and correlation (**b**) between 10-deacetyltaxol (10-DAT) content and mean of relative humidity; and relationship (**c**) and correlation (**d**) between 10-DAT content and mean of daylight length.

In this study, meteorological parameters including temperature, length of daylight, relative humidity and rainfall were obtained from PhyTalk system (Table 2). Subsequently, the contents of active compounds were examined for any correlation to climate changes. The data analyses showed that the paclitaxel level was negatively correlated with the mean of maximum temperature (R= −0.67), and negatively correlated with the mean of minimum temperature (R= −0.63) (Figure 1). The content of 10-DAB negatively depended on mean of minimum temperature and length of daylight with the correlation coefficients of R= −0.61 and R= −0.60, respectively (Figure 2). Besides, the 10-DAT level was negatively correlated with the length of daylight (R= −0.70) but positively correlated with the relative humidity (R= 0.70), as showed in Figure 3. These findings were in agreement with previous studies which showed that environment factors could influence taxoids content with higher yield in cool, moist and shady environments rather than warm, dry and sunny climates [37]. The taxoids content of *T. wallichiana* var. *mairei* in the shady slope was 1.8 times higher than those in sunny slope, meaning that the moderate shady condition is conducive to the accumulation of taxoids in Taxus species [38,39]. On the contrary, a study showed that the paclitaxel level was positively correlated to the temperature but the correlation coefficient was unsignificant [32], hence further investigation was needed to analyze the correlation between paclitaxel level and temperature.

Secondary metabolites function in plant defense against harsh environmental conditions and pathogens [32,40,41]. The five taxoids belonging to diterpenoids were important secondary metabolites in *T. wallichiana* var. *mairei*. Some diterpenoids such as colophony and resin are vital defense compounds [40], and thus taxoids are considered as defense compounds. In this study, the highest level of taxoid content emerged in winter and had a significant negative correlation with temperature and length of daylight, indicating that more taxoids were induced to adapt harsh environmental conditions. Therefore, it seems that certain stress measures could promote the taxoid content in *T. wallichiana* var. *mairei* before collection.

### 2.2. Seasonal Variations of the Flavonoid Content

The total and individual content of three flavonoids were also influenced by seasonal change in the pattern of seasonal dynamics similar to taxoids. The seasonal variation of flavonids content was presented in Table 1. Significantly higher content of total flavonoids (15.82 ± 1.45 mg·g^−1^) was observed in August, compared to the lowest level (3.27 ± 0.73 mg·g^−1^) recorded in March, which was consistent with the findings from other flavonoid-rich plants showing that summer season maximized the flavonoid content compared to the winter season [42]. For individual flavonoid, the highest and lowest levels of ginkgetin (0.42 ± 0.15 and 0.03 ± 0.00 mg·g^−1^) were observed in August and December, respectively. The content of quercetin was actually lower in relative terms, and the peak value of quercetin was 0.16 ± 0.00 mg·g^−1^ in June. Among the three individual flavonoids, amentoflavone showed the greatest variation of content. In addition, amentoflavone was lower in summer, and significantly higher content (0.80 ± 0.02 mg·g^−1^) was observed in February.

The content of flavonoids was also analyzed for any correlation to temperature, the length of daylight, relative humidity and rainfall. The investigation found that there was not any significant correlation between flavonoid compound levels and meteorological parameters. In other flavonoid containing-medical plants, the correlation between flavonoid compounds levels and climate parameters has been reported that warm climate and longer duration of daylight resulted in an increase in flavonoids content [43,44]. In particular, the flavonoids have been suggested to function as natural antioxidants, regulate plant growth and exhibit development adapted to hostile environments [42,45]. Summer is the season for plants to suffer stress from environmental variables such as high temperature and long daylight. It was reported that maximum content of flavonoids in *Cistus ladanifer* was also produced in summer, increasing approximately three- to four-fold with respect to the secretion measured in spring [46]. As seen in the present study, the highest content of flavonoids was produced during summer. These can further explain the highest level of flavonoids occurring in summer. Therefore, we can collect needles of *T. wallichiana* var. *mairei* in summer as the crude medicine of flavonoids.

### 2.3. Seasonal Variation of Polysaccharide Content

The results of the resonance light scattering (RLS) method revealed that polysaccharide content varied significantly during the course of a year. Seasonal variation of polysaccharide content showed the pattern with a peak value of 28.52 ± 0.57 mg·g^−1^ in September, which was two times higher than the lowest content of 9.39 ± 0.17 mg·g^−1^ in January. The seasonal variation pattern of polysaccharide content is partly resulted from the change of water-soluble polysaccharide (WSP) content in *Cyclocarya paliurus*, which showed the higher content from September to November, inferring that the biosynthesis of polysaccharides likely increased during maturity/senescence [47]. However, the lowest content of polysaccharides in *T. wallichiana* var. *mairei* was recorded in January, which differed from the change of WPS content in *Cyclocarya paliurus*, whereas the lower content were observed during rapid growing season from May to August [47].

**Figure 4:**
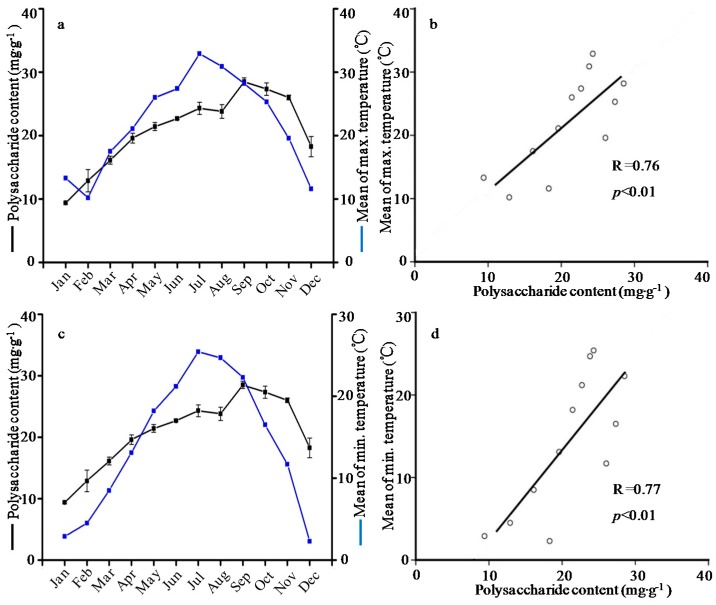
Relationship (**a**) and correlation (**b**) between polysaccharide content and mean of maximum temperature; and relationship (**c**) and correlation (**d**) between polysaccharide content and mean of minimum temperature.

Recent studies reported significantly positive correlations of polysaccharides content with altitude and sunshine hours [47]. Moreover, the constituents of polysaccharides in *Fructus jujubae* were correlated with climatic factors, the neutral carbohydrate content showed negative correlation with precipitation amount, and the uronic acid content was well correlated to the average annual temperature and frost-free period [48]. In the present study, there was a significantly positive correlation between polysaccharide content and temperature (Figure 4). Rainfall and length of daylight had a positive effect on polysaccharide accumulation, but the correlation was insignificant (*p* > 0.05). These discrepancies indicated that there were differences in the metabolic activity in various plant species.

## 3. Materials and Methods

### 3.1. Plant Materials and Chemicals

**Figure 5:**
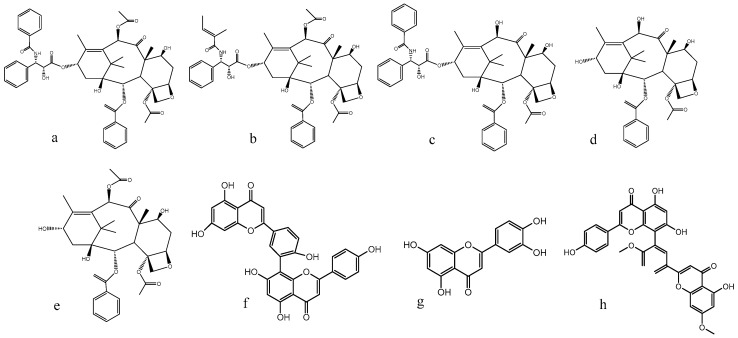
Structure of the five taxoids and three flavonoids. (**a**) paclitaxel; (**b**) cephalomannine; (**c**) 10-DAB; (**d**) 10-DAT; (**e**) baccatin III; (**f**) amentoflavone; (**g**) quercetin; and (**h**) ginkgetin.

All needle samples were collected twice a month (1st and 15th each month) in 2013 from 15-year old trees (12–16 year old trees were used for the production of herbal pieces) located at the campus of the Ningbo Institute of Technology, Zhejiang University (29°18′′ N/117°32′ E, Ningbo, China). Each time, the needles of three plants were collected at one site (middle crown position) and mixed to form a pool representing the individual. All samples were dried in the oven at 60 °C to a constant weight, grounded into powder using herbal pulverizer (FW 100, Tianjin Taisite Instrument Co. Ltd., Tianjin, China) and sieved through 250 μm filter. In addition, paclitaxel, 10-deacetylbaccatin III (10-DAB), baccatin III, cephalomannine, 10-deacetyltaxol (10-DAT), quercetin, amentoflavone, ginkgetin (Figure 5) and glucose standards (98% purity) were purchased from Shanghai Yuanye Bio-Technology (Shanghai, China). Acetonitrile and methanol (spectra analyzed grade) came from TEDIA Chemicals (Charlotte, NC, USA). All other chemicals and solvents in analytical grade were purchased from commercial sources. All samples prepared for UPLC were filtered through a 0.22 μm membrane filter before use.

### 3.2. Meteorological Parameter Collection

The PhyTalk system (version 2.4, PhyTech Ltd., Tel Aviv, Israel) was used for the monthly automatic collection of the meteorological parameters during 2013. The Phytomonitor system consists of environmental sensors and data collection, and a management system of the two sections. The signals from the PhyTalk system were recorded every 20 s and stored as 30 min average with an automatic data logger. Meteorological parameters including temperature, length of daylight, relative humidity and rainfall were measured simultaneously.

### 3.3. Preparation of Samples

#### 3.3.1. Preparation of Taxoid Sample

The ground needles (2 g) were extracted using 10 mL methanol with ultrasonic processing twice for 15 min at 60 °C. The extract was centrifuged at 4000× *g* for 10 min. The supernatant was combined and evaporated to dryness in a rotary evaporator at 60 °C. After that, the residue was resolved with 10 mL dichloromethane and partitioned with 2 mL water. Subsequently, the dichloromethane fraction was evaporated and suspended with 10 mL ethanol, and filtered through the 0.22 μm membrane [8].

#### 3.3.2. Preparation of Flavonoid Sample

The ground needles (2 g) were extracted with 10 mL methanol for 2 h at 50 °C. Then, the ultrasound-assisted extraction was performed for 15 min. This extraction process was repeated twice. The extracts were filtered with filter paper and collected. The mixture was concentrated to dryness by a rotary evaporator. The residue was resolved with 50 mL ethanol and filtered through a 0.22 μm membrane.

#### 3.3.3. Preparation of Polysaccharide Sample

The ground needles (5 g) were treated twice with ethanol for 12 h to remove lipids and pigments. Then, the defatted mixture was extracted twice with 100 mL distilled water for 4 h at 90 °C. The combined aqueous extracts were filtered and concentrated to 10 mL solution. After centrifugation at 4000× *g* for 10 min, the precipitate was dissolved in distilled water and diluted to 25 mL.

### 3.4. Determination of Sample Component Contents

#### 3.4.1. Determination of the Taxoid Content

**Figure 6:**
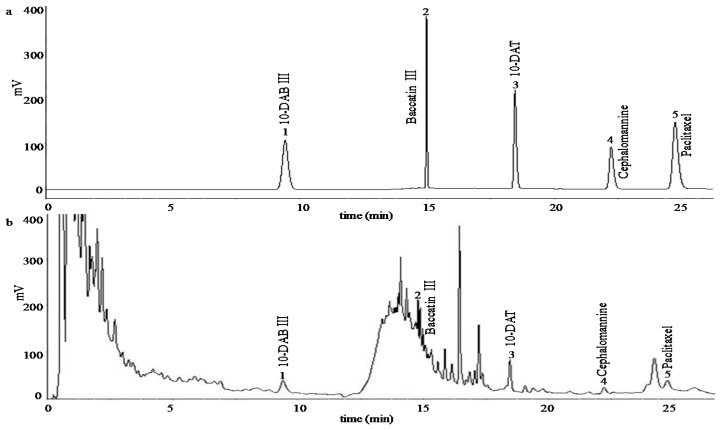
UPLC chromatogram of the five taxoids. (**a**): standard chromatogram; and (**b**): samples chromatogram. Chromatogram conditions were given in Section 3.4.1.

Chromatographic analysis was performed using the Agilent 1290 UPLC system with a ZORBAX Eclipse C18 column (2.1 × 150 mm, 1.8 μm) (Santa Clara, CA, USA). The mobile phase was composed of acetonitrile (A) and water (B) in gradient manner. The gradient was as follows: 0–10 min, 20% A; 10–14 min, 20%–40% A; 14–25 min, 40% A. The flow rate was 0.40 mL·min^−1^ and the column temperature was kept at 30 °C; the sample injection volume was 4 μL and scan wavelength was set at 254 nm (Figure 6) [49]. Linear regression analysis was performed by the external standard method. The calibration curves of the individual taxoid were constructed using a range of six concentrations of the standard. All taxoids showed excellent linearity (R^2^ > 0.99) in a relatively wide concentration range. The calibration curves were as follows: paclitaxel (*y* = 40*x* − 0.05, 0.005–0.20 mg·mL^−1^), 10-DAB (*y* = 40*x* − 0.04, 0.01–0.50 mg·mL^−1^), 10-DAT (*y* = 50*x* − 0.13, 0.005–0.20 mg·mL^−1^), cephalomannine (*y* = 40*x* − 0.02, 0.002–0.10 mg·mL^−1^), and baccatin III (*y* = 30*x* + 0.15, 0.01–0.50 mg·mL^−1^). The yields of paclitaxel, 10-DAB, 10-DAT, cephalomannine and baccatin III were quantitatively determined by the calibration curves, respectively.

#### 3.4.2. Determination of the Flavonoid Content

**Figure 7:**
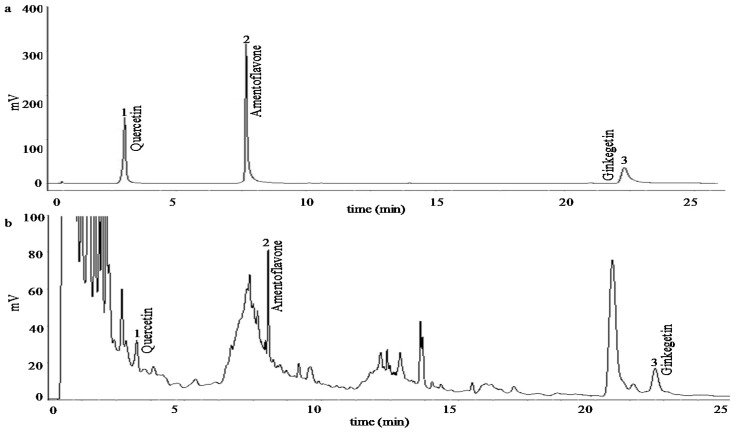
UPLC chromatogram of the three flavonoids. (**a**): standard chromatogram; and (**b**): samples chromatogram. Chromatogram conditions were given in Section 3.4.2.

The total flavonoids content was determined using a modified colorimetric method, calculated by a rutin standard curve (*y* = 9.04*x* − 0.03) [50]. Chromatographic analysis of the quercetin, amentoflavone, ginkgetin was also conducted on the UPLC system. A ZORBAX Eclipse C18 column (2.1 × 150 mm, 1.8 μm) was used and eluted with a gradient of acetonitrile (A) and water (B) at a flow rate of 0.40 mL·min^−1^ and a temperature of 30 °C. The elution program was set as follows: 0–5 min, 20% A; 5–6 min, 20%–30% A; 6–10 min, 30% A; 10–12 min, 30%–40% A; 12–15 min, 40%–60% A; 15–18 min, 85% A; 18–20 min, 100% A; and 20–25 min, 5% A. Detection was conducted at a wavelength of 360 nm (Figure 7). A series of standards (*n* = 6) in the range of 0.001–0.05, 0.002–0.10 and 0.002–0.20 mg·mL^−1^ for amentoflavone, quercetin and ginkgetin, respectively, were prepared in methanol, giving linear calibration curves *y* = 40*x* − 0.01, R^2^ = 0.99; *y* = 80*x* − 0.11, R^2^ = 0.99 and *y* = 40*x* − 0.02, R^2^ = 0.99 for them, respectively. The yields of amentoflavone, quercetin and ginkgetin were quantitatively determined by the calibration curves, respectively.

#### 3.4.3. Determination of the Polysaccharides

For the experiment, 1 mL of cetylpyridinium chloride (CPC) (1 mM), 0.50 mL NaOH (1 M) and 2 mL polysaccharides were added into a tube and mixed rapidly. Then, the mixed solution was removed from the test tube to a cuvette for RLS. The RLS spectra were obtained by scanning simultaneously excitation and emission monochromators of the spectrofluorometer from 300 to 800 nm. The enhanced RLS intensity of CPC was represented as △*I*_RLS_ = *I*_RLS_ − *I*^0^_RLS_, where *I*_RLS_ and *I*^0^_RLS_ were the intensities of the systems with and without polysaccharides [51].

### 3.5. Statistical Analyses

All resulting data were presented as the mean ± standard error of three replications. SPSS version 16.0 (IBM, Armonk, NY, USA) was used for statistical analysis. ANOVA was conducted to compare the compound content every month. A correlation was considered to be significant when *p* < 0.05. The results of ANOVA showed the significant differences at the 0.05 significance level for multiple comparisons among the different months. Correlation between the content of detected compounds and climatic parameters were determined using Pearson’s correlation analysis.

## 4. Conclusions

The quantitative analysis of UPLC showed that the highest content of taxoids (1.77 ± 0.38 mg·g^−1^) was found in January. On the contrary, the maximum content of total flavonoids obtained in the spectrophotometry (15.82 ± 1.45 mg·g^−1^) was observed in August. The polysaccharide accumulation reached a value of 28.52 ± 0.57 mg·g^−1^ in September. Moreover, the mean of the minimum temperature and length of daylight were significantly negatively correlated to 10-DAB content, while 10-DAT level showed significant correlation with length of daylight (R = −0.70) and relative humidity (R = 0.70). Temperature had a significantly negative effect on paclitaxel content and a significantly positive effect on polysaccharides.

## Figures and Tables

**Table 1:** Variation of taxoids, flavones and polysaccharides contents in *T. wallichiana* var. *mairei*.

Collection Date	Content of Investigated Taxoids (mg·g^−1^)						Content of Investigated Flavonoids (mg·g^−1^)				Content of Investigated Polysaccharides (mg·g^−1^)
	10-DAB	Baccatin	10-DAT	Cephalomannine	Paclitaxel	Sum	Quercetin	Amentoflavone	Ginkgetin	Total Flavonoids	Polysaccharides
January	0.76 ± 0.06a	0.47 ± 0.12b	0.18 ± 0.00abc	0.10 ± 0.02bc	0.26 ± 0.05abc	1.77 ± 0.38a	0.12 ± 0.01ab	0.66 ± 0.01ab	0.15 ± 0.05bcd	3.91 ± 0.69de	9.39 ± 0.17j
February	0.41 ± 0.04bcd	0.54 ± 0.13ab	0.23 ± 0.08ab	0.12 ± 0.00ab	0.40 ± 0.08a	1.71 ± 0.51ab	0.10 ± 0.01abc	0.80 ± 0.02a	0.20 ± 0.09bc	5.97 ± 0.87cde	12.89 ± 1.76i
March	0.32 ± 0.06bcd	0.35 ± 0.01bc	0.16 ± 0.05abc	0.12 ± 0.01ab	0.25 ± 0.04bcd	1.19 ± 0.14bcd	0.10 ± 0.02abc	0.45 ± 0.02abc	0.12 ± 0.03bcd	3.27 ± 0.73e	16.12 ± 0.63h
April	0.18 ± 0.07d	0.32 ± 0.01bc	0.13 ± 0.07abc	0.08 ± 0.04bc	0.13 ± 0.04cd	0.84 ± 0.19de	0.06 ± 0.01bc	0.30 ± 0.00bc	0.11 ± 0.01bcd	4.30 ± 0.18de	19.59 ± 0.77fg
May	0.48 ± 0.03b	0.43 ± 0.08bc	0.16 ± 0.04abc	0.10 ± 0.00bc	0.26 ± 0.04abc	1.44 ± 0.06abc	0.14 ± 0.05a	0.49 ± 0.02abc	0.08 ± 0.00cd	13.75 ± 1.64a	21.43 ± 0.62ef
June	0.46 ± 0.03bcd	0.47 ± 0.13b	0.26 ± 0.03a	0.12 ± 0.04ab	0.19 ± 0.02bcd	1.50 ± 0.51abc	0.16 ± 0.00a	0.31 ± 0.00bc	0.11 ± 0.05bcd	13.06 ± 0.73a	22.70 ± 0.13de
July	0.17 ± 0.06d	0.45 ± 0.05bc	0.14 ± 0.02abc	0.09 ± 0.00bc	0.18 ± 0.00bcd	1.03 ± 0.11cde	0.14 ± 0.02a	0.38 ± 0.00bc	0.13 ± 0.00bcd	11.26 ± 1.22b	24.30 ± 0.96cd
August	0.26 ± 0.02bcd	0.39 ± 0.08bc	0.10 ± 0.06bc	0.12 ± 0.01ab	0.21 ± 0.02bcd	1.09 ± 0.13cde	0.10 ± 0.00abc	0.45 ± 0.01abc	0.42 ± 0.15a	15.82 ± 1.45a	23.81 ± 1.08d
September	0.19 ± 0.13cd	0.20 ± 0.02c	0.07 ± 0.02c	0.04 ± 0.00c	0.10 ± 0.04d	0.61 ± 0.08d	0.04 ± 0.00c	0.46 ± 0.03abc	0.16 ± 0.03bcd	9.72 ± 0.42bc	28.52 ± 0.57a
October	0.33 ± 0.07bcd	0.56 ± 0.12ab	0.14 ± 0.05abc	0.13 ± 0.00ab	0.23 ± 0.00bcd	1.39 ± 0.04abc	0.12 ± 0.01ab	0.30 ± 0.00bc	0.23 ± 0.10bc	8.66 ± 1.40bc	27.33 ± 0.98ab
November	0.51 ± 0.03b	0.78 ± 0.22a	0.17 ± 0.01abc	0.08 ± 0.07bc	0.17 ± 0.06bcd	1.71 ± 0.31ab	0.06 ± 0.01bc	0.20 ± 0.00c	0.27 ± 0.10b	7.02 ± 0.93bcd	26.03 ± 0.37bc
December	0.47 ± 0.02bc	0.57 ± 0.04ab	0.12 ± 0.01abc	0.18 ± 0.03a	0.30 ± 0.03ab	1.64 ± 0.01ab	0.06 ± 0.00bc	0.11 ± 0.00c	0.03 ± 0.00d	7.18 ± 1.62bcd	18.29 ± 1.59g

Note: The same lower-case letter indicates a non-significant difference (*p* > 0.05); different lower-case letters indicate a significant difference (*p* < 0.05).

**Table 2:** Growth conditions of *T. wallichiana* var. *mairei* during 2013.

Month	Related Season	Mean of Maximum Temperature (°C)	Mean of Minimum Temperature (°C)	Daylight (min)	Relative Humidity (%)	Total Rainfall (mm)
December	Winter	11.64	2.31	173.1	64.20	114.32
January		13.26	2.94	122.76	74.77	34.07
February		10.25	4.46	46.02	80.18	89.16
March	Spring	17.47	8.53	171.04	69.80	114.50
April		21.13	13.06	192.83	60.03	112.52
May		26.02	18.18	192.38	74.53	73.59
June	Summer	27.39	21.23	100.60	82.36	324.20
July		32.88	25.40	338.29	63.32	69.28
August		30.87	24.73	263.14	67.23	251.64
September	Autumn	28.20	22.29	223.75	71.02	32.89
October		25.29	16.55	161.32	73.37	424.00
November		19.57	11.68	178.30	67.40	42.64
